# Assessment of neurocognitive impairment in HIV-infected patients from the Regional Center Mureş

**DOI:** 10.1186/1471-2334-14-S4-P37

**Published:** 2014-05-29

**Authors:** Mariana Georgescu, Carmen Chiriac, Erzsébet Iringó Zaharia-Kézdi, Nina Șincu

**Affiliations:** 1 Infectious Diseases Clinic I, Tîrgu Mureş County Clinic Hospital, Tîrgu Mureş, Romania

## 

Neurocognitive assessment in HIV-infected patients in Regional Center Mureş was held between 01.01.2013-31.12.2013 on a group of 26 patients. Out of the 26 patients recruited and evaluated, all were included in the national database and 6 patients were recommended for comprehensive assessment at the “Victor Babeş” Hospital - Bucharest. Inclusion criteria: patients born between 1987-1991, at least 8 years of education, who had not been institutionalized, without psychiatric conditions.

We used the following methods for neurocognitive screening of patients included in our study: International HIV Dementia Scale, Simioni questionnaire, Beck Depression Inventory, Patient’s Assessment of Own Functioning Inventory (PAOFI).

Preliminary psychological evaluation in 26 patients showed no evidence of neurocognitive deficit. Out of the 6 patients who underwent comprehensive neurocognitive testing, 1 patient was diagnosed with neurocognitive deficit, obtaining lower values than the group’s average for most tested areas.

It is necessary to validate a tool to identify the neurocognitive changes caused by HIV. The final results will be given by the National Screening Program.

